# Mental health status and quality of life in close contacts of COVID-19 patients in the post-COVID-19 era: a comparative study

**DOI:** 10.1038/s41398-021-01623-0

**Published:** 2021-10-02

**Authors:** Yan-Jie Zhao, Shu-Fang Zhang, Wen Li, Ling Zhang, Teris Cheung, Yi-Lang Tang, Chee H. Ng, Bing-Xiang Yang, Yu-Tao Xiang

**Affiliations:** 1 Unit of Psychiatry, Department of Public Health and Medicinal Administration, & Institute of Translational Medicine, Faculty of Health Sciences, University of Macau, Macao SAR, China; 2 Centre for Cognitive and Brain Sciences, University of Macau, Macao SAR, China; 3 Institute of Advanced Studies in Humanities and Social Sciences, University of Macau, Macao SAR, China; 4 Research Center for Psychological and Health Sciences, China University of Geosciences, Wuhan, Hubei China; 5 Department of Psychiatry, Wuhan Mental Health Center, Wuhan, Hubei Province China; 6 The National Clinical Research Center for Mental Disorders & Beijing Key Laboratory of Mental Disorders Beijing Anding Hospital & the Advanced Innovation Center for Human Brain Protection, Capital Medical University, School of Mental Health, Beijing, China; 7 School of Nursing, Hong Kong Polytechnic University, Hong Kong SAR, China; 8 Department of Psychiatry and Behavioral Sciences, Emory University, Atlanta, GA USA; 9 Mental Health Service Line, Atlanta VA Medical Center, Decatur, GA USA; 10 Department of Psychiatry, The Melbourne Clinic and St Vincent’s Hospital, University of Melbourne, Richmond, VIC Australia; 11 School of Health Sciences, Wuhan University, Wuhan, Hubei China

**Keywords:** Scientific community, Psychiatric disorders

## Abstract

Close contacts of those with COVID-19 (CC) may experience distress and long-lasting mental health effects. However, the mental health status and quality of life (QOL) in CC have not been adequately examined. This study examined the mental health status and QOL in CC during the post-COVID-19 period. This cross-sectional study comprised 1169 CC and 1290 who were non-close contacts (non-CC). Demographic data were collected; depression, fatigue, post-traumatic stress symptoms (PTSS) and QOL were assessed using the Patient Health Questionnaire - 9 items (PHQ-9), fatigue numeric rating scale, Post-Traumatic Stress Disorder Checklist - 17 items (PCL-17), and the World Health Organization Quality of Life Questionnaire - brief version (WHOQOL-BREF), respectively. Analysis of covariance was used to compare depressive symptoms, QOL, fatigue, and PTSS between the CC and non-CC groups. Multiple logistic regression analyses were performed to determine the independent correlates for depression, fatigue, PTSS, and QOL in the CC group. Compared to the non-CC group, the CC group reported significantly more severe depression (*F*_(1, 2458)_ = 5.58, *p* = 0.018) and fatigue (*F*_(1, 2458)_ = 9.22, *p* = 0.002) in the post-COVID-19 period. No significant differences in PTSS and QOL between the CC and non-CC groups were found (*F*_(1, 2458)_ = 2.93, *p* = 0.087 for PTSS; *F*_(1, 2458)_ = 3.45, *p* = 0.064 for QOL). In the CC group, younger age, financial loss due to COVID-19, and perception of poor or fair health status were significantly associated with depression and fatigue, while frequent use of mass media was significantly associated with fatigue. In conclusion, close contacts of COVID-19 patients experienced high levels of depression and fatigue in the post-COVID-19 period. Due to the negative effects of depression and fatigue on daily functioning, early detection and timely interventions should be provided to this neglected population.

## Introduction

At the end of 2019, coronavirus disease 2019 (COVID-19) was first reported in Wuhan, Hubei province of China and subsequently was also found in other parts of the world [1, 2]. Due to its fast transmission rate, the World Health Organization (WHO) declared the COVID-19 outbreak a pandemic on March 11, 2020 [3]. By the middle of March 2021, there were over 117 million COVID-19 cases globally with 2.6 million deaths. At the same time, over 66 million people have recovered from this disease [4].

Although research has focused on patients with COVID-19 [5, 6], few studies have reported on the close contacts of COVID-19 patients (CC hereafter). Close contacts are at high risk of contracting COVID-19 infection [7–10]. Further, restrictions imposed on them, including mandatory quarantine in designated places or at home and frequent virus testing [11, 12], can also increase the risk of physical and mental health problems.

According to previous studies, CC usually refer to people who is within 6 feet (or 2 m) of an infected person for a total of ≥15 min, or who live in the same household or shared accommodation with an infected person, or who travel in the same vehicle or an airplane with an infected person, or who have direct contact with body fluids or secretions of an infected person (e.g., was coughed or sneezed on) [13–19]. The number of CC is difficult to estimate or track; previous studies found that one confirmed COVID-19 case could have up to 44 close contacts on average [20–25].

Given the high number of CC and the adverse impact of the pandemic on them, it is important to examine their mental health status and quality of life (QOL). In the past year, studies have found that CC had increased risk of mental health problems such as anxiety, depression, and psychological distress during the COVID-19 pandemic [26–28]. In addition, QOL has gained increasing attention as an important health outcome in clinical practice and research during the pandemic [29]. An Italian study found that the frontline healthcare staff reported lower QOL than their non-frontline counterparts [30]. Furthermore, long-term negative mental health impact of biological disasters (e.g., outbreak of infectious diseases) may occur in various populations even after the outbreak is controlled, with different clinical features compared to those during the outbreak [31–34]. A longitudinal study on severe acute respiratory syndrome (SARS) found that healthcare workers who cared for infected patients had higher stress level at the 1-year follow-up after the SARS outbreak compared to non-healthcare workers [31]. However, no studies have examined the mental health status and life quality in CC in the post COVID-19 period.

For 76 days, the city of Wuhan, the epicenter of the COVID-19 outbreak in China, was under “lockdown,” with travel restrictions and other public health and administrative measures until the 8 April 2020 [35, 36]. The “post-COVID-19 era” in this study refers to the period after the lockdown policy and related public restrictions in Wuhan were lifted on April 8, 2020 after no new cases were reported for 19 days in Wuhan [37, 38]. Additionally, during the 12-month period between April 8, 2020 and April 8, 2021, only 350 new cases were diagnosed in Hubei province [37], indicating that there was no further serious outbreak after the lockdown policy was canceled. Wuhan is not only the first epicenter of the COVID-19 outbreak globally but also the first major city where the outbreak was rapidly brought under control; therefore, Wuhan is one of the most suitable areas to conduct “post-COVID-19 era”-related research.

This study examined the mental health status, such as depression, fatigue, post-traumatic stress symptoms (PTSS), and QOL in CC during the post-COVID-19 period. Based on previous relevant findings [31–34], we hypothesized that the CC group have higher levels of depression, fatigue, PTSS, and lower QOL than the non-CC group.

## Methods

### Study setting and participants

This was a cross-sectional, comparative study conducted during the post-COVID-19 period between May 25, 2020 and June 18, 2020 in Wuhan, Hubei province, China. Following previous studies [39–41], to minimize the risk of infection, participants were recruited and assessed online using the WeChat-based QuestionnaireStar program (Changsha Haoxing Information Technology Co., Ltd., Changsha, China) based on snowball sampling. A QuestionnaireStar Quick Response (QR) code linked to the invitation and assessments was disseminated by study team members, their colleagues, and friends who worked and lived in Wuhan via WeChat, which is the most popular social network application in China with around 1.2 billion monthly active users [42]. Persons who completed the assessments in this study were encouraged to invite people around them to participate in this study. The QuestionnaireStar program has been widely used in observational studies during the COVID-19 pandemic.

To be eligible, participants needed to meet the following criteria: (1) age ≥18 years; (2) able to read Chinese and understand the purpose and contents of the assessments; (3) not infected with COVID-19 during the pandemic; and (4) provided online electronic informed consent. Participants were divided into two groups: CC group and control group (non-CC hereinafter). Close contacts were defined as individuals who had family members, colleagues, close friends, or neighbors infected with COVID-19; this practical definition was widely used in clinical practice [11]. The study protocol was approved by the ethics committee of Beijing Anding Hospital, Capital Medical University.

### Assessment tools

An electronic data collection form was designed to collect demographic and clinical data, including age, gender, education level, occupation, place of residence, living in urban or rural areas, living status (alone or with family members), frequency of mass media use, financial loss due to the COVID-19 pandemic, and perception of financial and health status. They were asked whether they had family members, colleagues, close friends, or neighbors infected with COVID-19 and whether they had previously been infected with COVID-19.

Severity of depressive symptoms (depression hereafter) was assessed using the Chinese version of the Patient Health Questionnaire - 9 items (PHQ-9), which consists of 9 items and each scored from 0 (not at all) to 3 (almost every day) [43, 44]. A higher score represents more severe depression [45]. The psychometric properties of PHQ-9 Chinese version have been validated in Chinese populations [46, 47]. Participants were classified as “having depression” if their PHQ-9 total score was ≥5 [45]. Overall QOL was assessed with the first two items of the World Health Organization Quality of Life Questionnaire - brief version (WHOQOL-BREF) [48, 49], with a higher score representing higher overall QOL [50]. Fatigue was assessed using the 11-point fatigue numeric rating scale, ranging from 0 (no fatigue) to 10 (the worst fatigue you can imagine) [51–53]. Fatigue total score ≥4 was considered as “having clinically significant fatigue” (“having fatigue” hereinafter) [54]. PTSS was assessed using the Chinese version of the Post-Traumatic Stress Disorder Checklist - 17 items (PCL-17) [55, 56]. Generally, Chinese people with psychiatric disorders tend to express their mental health problems in terms of physical symptoms [57–59]. Therefore, fatigue is not only a physical symptom but also a very common somatic symptom of psychiatric disorders. The PCL-17 is a 5-point Likert scale, with each item scoring from 1 (not at all) to 5 (extremely) in three domains: intrusion, avoidance/numbing, and hyperarousal. The Chinese version of the PCL-17 has been shown to have satisfactory psychometric properties [56].

### Statistical analysis

All the data analyses were conducted using Statistical Analysis System (SAS), University Edition (SAS Institute Inc., Cary, NC, USA). In univariable analyses, the demographic and clinical characteristics between close contacts and non-close contacts were compared using independent two-sample *t* tests, Wilcoxon rank-sum tests, and chi-square tests as appropriate. Analysis of covariance (ANCOVA) was used to compare depressive symptoms, overall QOL, fatigue, and PTSS between the CC and the non-CC groups after adjusting for variables that significantly differed in univariable analysis (confounders hereafter). Multiple logistic regression was applied to determine the independent demographic and clinical correlates for depression, fatigue, PTSS, and QOL among CC if they were significantly different from non-CC. Two-sided *p* values <0.05 were considered as statistically significant.

Around half of participants in the CC group were medical workers. As the high proportion of medical workers might bias the results, a post hoc sensitivity analysis was conducted by excluding medical workers to examine whether this group could significantly affect the original results of logistic regression analyses. In the post hoc sensitivity analyses, similar logistic regression conducted in the whole sample were repeated, and then the results were compared with the original ones in the whole sample.

## Results

In total, 2614 were invited to participate in this study; 2459/2614 (94.1%) participants fulfilled the study entry criteria and completed the assessments, including 1169 CC and 1290 non-CC. Table 1 shows the basic demographic data of the participants. There were significant differences between the CC and non-CC groups in terms of age, occupation, place of residence, living area (urban or rural), frequency of mass media use, financial loss due to COVID-19, and perceived economic and health status (all *p* values <0.05). After adjusting for confounders, close contacts still had more severe depression (*F*_(1, 2458)_ = 5.58, *p* = 0.018) and fatigue (*F*_(1, 2458)_ = 9.22, *p* = 0.002), while no significant difference in PTSS and overall QOL between the two groups were found (*F*_(1, 2458)_ = 2.93, *p* = 0.087) for PTSS; *F*_(1, 2458)_ = 3.45, *p* = 0.064 for QOL).

**Table 1:** Demographic and clinical characteristics of the whole study sample.

Variables	Non-close contacts (*N* = 1290)		Close contacts (*N* = 1169)		Univariable analysis			ANCOVA^c^		
	*n*	%	*n*	%	*χ*^2^	*df*	*p*	*F*	*df*	*p*
Male gender	331	25.7	298	25.5	0.01	1	0.92	—	—	—
College and above	1168	90.5	1081	92.5	2.92	1	0.09	—	—	—
Occupation					139.44	2	<0.001	—	—	—
Medical workers	360	27.9	598	51.2						
Other occupation	521	40.4	324	27.7						
Not recorded or unemployed	409	31.7	247	21.1						
Place of residence					563.44	2	<0.001	—	—	—
Wuhan city	491	38.1	967	82.7						
Other areas in Hubei province	346	26.8	162	13.9						
Other provinces	453	35.1	40	3.4						
Living in urban (vs. rural)	1057	81.9	1096	93.8	78.60	1	<0.001	—	—	—
Living with families (vs. living alone)	1085	84.1	1007	86.1	2.00	1	0.16	—	—	—
Frequent use of mass media	952	73.8	963	82.4	26.20	1	<0.001	—	—	—
Financial loss due to COVID-19					50.09	2	<0.001	—	—	—
None or minimal	410	31.8	234	20.0						
Moderate	707	54.8	706	60.4						
Significant	173	13.4	229	19.6						
Perception of financial status					9.69	2	0.008	—	—	—
Poor	245	19.0	175	15.0						
Fair	965	74.8	936	80.1						
Good	80	6.2	58	5.0						
Perception of health status					33.14	2	<0.001	—	—	—
Poor	14	1.1	19	1.6						
Fair	451	35.0	536	45.9						
Good	825	64.0	614	52.5						
	**Mean**	**SD**	**Mean**	**SD**	***t*/*Z***	**df**	***p***	***F***^c^	**df**	***p***
Age (years)	33.7	11.4	37.2	10.1	8.19	2455.6^a^	<0.001	—	—	—
PHQ-9 total score	4.8	4.9	5.5	5.0	4.52	—^b^	<0.001	5.58	1	0.018
Overall QOL	6.7	1.3	6.4	1.3	-5.59	2399.1^a^	<0.001	3.45	1	0.064
Fatigue score	3.8	2.3	4.4	2.3	6.29	—^b^	<0.001	9.22	1	0.002
PCL-17 total score	22.7	7.7	24.4	8.3	5.08	2387.8^a^	<0.001	2.93	1	0.087

*ANCOVA* analysis of covariance, *COVID-19* coronavirus disease 2019, *df* degree of freedom, *SD* standard deviation, *QOL* quality of life, *PHQ-9* patient health questionnaire—9 items, *PCL-17* post-traumatic stress disorder checklist—17 items.

^a^Satterthwaite corrected.

^b^Wilcoxon rank-sum test.

^c^Adjusted for age, occupation, place of residence, living area (urban or rural), frequent use of mass media, financial loss due to COVID-19, health perception.

In the CC group, 50.2% (95% confidence interval (CI): 47.3–53.1%) were classified as “having depression,” while the corresponding figure was 43.9% (95% CI: 41.2–46.6%) among non-CC. The prevalence of fatigue was 63.8% (95% CI: 61.1–66.6%) and 54.3% (95% CI: 51.5–57.0%) in the CC and non-CC groups, respectively. Multiple logistic regression analysis revealed that older age (odds ratio (OR) = 0.97, 95% CI: 0.96–0.98), significant financial loss during COVID-19 (OR = 2.0, 95% CI: 1.4–3.0), and perception of poor/fair health (OR = 3.9, 95% CI: 3.0–5.0) were significantly associated with depression in CC. In contrast, older age (OR = 0.97, 95% CI: 0.96–0.99), frequent use of mass media (OR = 1.4, 95% CI: 1.02–1.97), financial loss (OR = 1.6, 95% CI: 1.2–2.2 for moderate financial loss; OR = 2.6, 95% CI: 1.7–3.9 for significant financial loss), and perception of poor/fair health (OR = 3.3, 95% CI: 2.5–4.3) were significantly associated with fatigue in the CC group (Table 2).

**Table 2:** Independent correlates of depression and fatigue among close contacts (*N* = 1169).

Variables	Non-depression (*N* = 582)		Depression (*N* = 587)		Multiple logistic regression			Non-fatigue (*N* = 423)		Fatigue (*N* = 746)		Multiple logistic regression		
	Mean	*SD*	Mean	*SD*	*p*	*OR*	95% *CI*	Mean	*SD*	Mean	*SD*	*p*	*OR*	95% *CI*
Age (years)	38.4	10.4	36.1	9.6	<0.001	0.97	0.96–0.98	38.3	10.7	36.6	9.6	<0.001	0.97	0.96–0.99
	***n***	**%**	***n***	**%**				***n***	**%**	***n***	**%**			
Female	428	73.5	443	75.5	0.89	1.0	0.7–1.3	319	75.4	552	74.0	0.12	0.8	0.6–1.1
Occupation														
Medical workers	298	51.2	300	51.1	—	—	—	196	46.3	402	53.9	—	—	—
Other occupations	168	28.9	156	26.6	0.29	1.2	0.9–1.6	131	31.0	193	25.9	0.28	0.8	0.6–1.1
Not recorded and unemployed	116	19.9	131	22.3	0.39	1.2	0.8–1.6	96	22.7	151	20.2	0.43	0.9	0.6–1.2
Place of residence														
Wuhan city	485	83.3	482	82.1	0.96	1.0	0.5–2.0	332	78.5	635	85.1	0.54	1.2	0.6–2.5
Other areas in Hubei province	78	13.4	84	14.3	0.60	1.2	0.6–2.6	75	17.7	87	11.7	0.68	0.9	0.4–1.8
Other provinces	19	3.3	21	3.6	—	—	—	16	3.8	24	3.2	—	—	—
Living in urban areas (vs. rural)	548	94.2	548	93.4	0.47	0.8	0.5–1.4	390	92.2	706	94.6	0.18	1.4	0.9–2.4
Frequent use of mass media	475	81.6	488	83.1	0.34	1.2	0.8–1.6	338	79.9	625	83.8	0.038	1.4	1.02–1.97
Financial loss due to COVID-19														
None or minimal	133	22.9	101	17.2	—	—	—	117	27.7	117	15.7	—	—	—
Moderate	361	62.0	345	58.8	0.35	1.2	0.8–1.6	250	59.1	456	61.1	0.002	1.6	1.2–2.2
Significant	88	15.1	141	24.0	<0.001	2.0	1.4–3.0	56	13.2	173	23.2	<0.001	2.6	1.7–3.9
Poor or fair health perception (vs. good)	189	32.5	366	62.4	<0.001	3.9	3.0–5.0	128	30.3	427	57.2	<0.001	3.3	2.5–4.3

*SD* standard deviation, *OR* odds ratio, *CI* confidential interval.

The results of post hoc sensitivity analysis (Supplementary Table 1) were similar to the original results achieved in the CC group (Table 2), indicating that the high proportion of medical workers did not significantly affect the results.

## Discussion

To the best of our knowledge, this was the first study that investigated the mental health status and QOL among CC in the post-COVID-19 period. Based on this study sample recruited from the previous epicenter of COVID-19 and using validated assessment tools, we found that, after adjusting for potential confounders, CC experienced significantly higher rates of depression and fatigue symptoms compared to non-CC. As no previous studies compared mental health status between the CC and non-CC groups in the post-COVID-19 period, direct comparisons with other studies could not be made. However, other studies on mental health status of CC during the COVID-19 pandemic found that this population were more likely to report higher prevalence of depression compared to community-dwelling residents and health professionals who were not close contacts [28, 60].

Several factors could contribute to more severe depression and fatigue in the CC compared to the non-CC group. First, CC are likely to experience fear, anxiety, and frustration due to their increased risk of infection [61, 62]. Second, given that their close ones were infected with COVID-19, and that physical and psychiatric comorbidities were common in COVID-19 survivors [63–66], CC are more likely to experience distress. Third, due to the extended period (14–21 days) of mandatory quarantine [11, 12], CC are likely to face more financial burden and increased risk of mental health problems [67]. Finally, stigma and discrimination associated with COVID-19, as well as social isolation, are risk factors for depression, anxiety, and fatigue [68–71]. All these factors could increase the risk of depression, which could further contribute to fatigue and other somatic symptoms [72, 73]. Furthermore, the proportion of medical workers in the CC group (51.2%) was much higher than that in the non-CC group (27.9%) in this study. Previous studies have found a high level of fatigue and burnout among healthcare workers during the COVID-19 outbreak [74–79], which also contributed to the higher prevalence of fatigue in the CC group.

The symptoms of depression and fatigue in the CC group were significantly associated with certain demographic and clinical correlates. Previous studies found mental health problems such as depression, anxiety, and psychological distress were more common in younger people during the COVID-19 pandemic [80–85]. Quarantine and other preventive measures during the pandemic may particularly affect the social and physical activities in younger people [86, 87]; in addition, younger people may have reduced resilience and coping mechanisms [88]. Consequently, they may be more likely to have depressive and fatigue symptoms as we found in this study. Financial loss due to COVID-19 was significantly associated with higher risk of depression and fatigue among close contacts, which confirms previous findings [89–91].

In this study, more frequent use of mass media was an independent correlate of more severe fatigue (not depression) among close contacts. During the pandemic and quarantine period, close contacts facing quarantine and social isolation usually spend more time on social media such as smartphone and the Internet. This could further reduce physical exercises and social communications with others, which could increase the risk factors of chronic fatigue [92–94]. In this study, perception of poor/fair health was associated with higher risk of both depression and fatigue in CC. The relationship between subjective health perception and depression/fatigue could be bidirectional. On the one hand, some studies found that perception of poor or moderate health was related to more severe depression in nursing students, older people, and primary care patients [95–98] and was related to fatigue in the general population and students with school-year employment [99, 100]. On the other hand, poor perception of health could lead to health-related anxiety and hypochondria, which may further result in depression, headaches, insomnia, and even suicidal ideation [101–103]. Another longitudinal study found that perception of good health was a predictor of less severe fatigue in worn-out employees [104]. One study found that fatigue played a causal role in the subjective perception of health [105].

The strengths of this study included the large sample size and the focus on CC in the pandemic epicenter during the post-COVID-19 period. In addition, an online survey was used, which could ensure anonymity. However, there were several methodological limitations. First, due to the cross-sectional design, no causal relationships between mental health status and other variables could be established. Second, due to logistical reasons, snowball, rather than random sampling, was used, which could lead to selection bias. In addition, around half of participants in the CC group were medical workers. However, post hoc sensitivity analyses did not find that the high proportion of medical workers significantly influence the original results. Third, certain important factors related to mental health of close contacts were not collected in this study, such as different types of close contacts (e.g., household contacts, work contacts, and social contacts [106]), social supports, and quarantine history and quarantine duration of the close contacts. Fourth, information on chronic or major diseases in medical records was not collected, although this could provide more precise information on participants’ physical health than their perception of health. Fifth, the potential impact of death or post-COVID-19 disabilities of COVID-19 patients could have an important impact on the mental health status of CC, but the relevant information was not collected in this study.

In conclusion, close contacts experienced high levels of depression and fatigue in post-COVID-19 period, particularly in those who were younger, had experienced financial loss due to COVID-19, and had perception of poor or fair health status. Considering the negative effects of depression and fatigue on QOL and daily functioning, early detection and timely intervention should be provided to this neglected population.

## Supplementary information

Supplementary Table 1. Post-hoc sensitivity analysis: independent correlates of depression and fatigue among close contacts after excluding medical workers (N=571)
