# Supplementary material for: Mental health status and quality of life in close contacts of COVID-19 patients in the post-COVID-19 era: a comparative study
Source: Transl Psychiatry. 2021 Oct 2;11:505. doi: 10.1038/s41398-021-01623-0 (PMC8487227; doi:10.1038/s41398-021-01623-0)
Supplement: Supplementary file 1 — Supplementary Table 1. Post-hoc sensitivity analysis: independent correlates of depression and fatigue among close contacts after excluding medical workers (N=571) [file 41398_2021_1623_MOESM1_ESM.docx]

Supplementary Table 1. Post-hoc sensitivity analysis: independent correlates of depression and fatigue among close contacts after excluding medical workers (N=571)

| Variables | Non depression  (N=284) | | Depression  (N=287) | | Multiple logistic regression | | | Non fatigue  (N=227) | | Fatigue  (N=344) | | Multiple logistic regression | | |
| --- | --- | --- | --- | --- | --- | --- | --- | --- | --- | --- | --- | --- | --- | --- |
|  | Mean | *SD* | Mean | *SD* | *p* | *OR* | *95% CI* | Mean | *SD* | Mean | *SD* | *p* | *OR* | *95% CI* |
| Age (years) | 38.8 | 10.8 | 37.1 | 10.6 | 0.007 | 0.98 | 0.96-0.99 | 38.4 | 11.1 | 37.6 | 10.4 | 0.052 | 0.98 | 0.97-1.00 |
|  | *n* | *%* | *n* | *%* |  |  |  | *n* | *%* | *n* | *%* |  |  |  |
| Female | 192 | 67.6 | 201 | 70.0 | 0.72 | 1.1 | 0.7-1.6 | 158 | 69.6 | 235 | 68.3 | 0.60 | 0.9 | 0.6-1.3 |
| Place of residence |  |  |  |  |  |  |  |  |  |  |  |  |  |  |
| Wuhan city | 208 | 73.2 | 204 | 71.1 | 0.80 | 1.1 | 0.5-2.2 | 153 | 67.4 | 259 | 75.3 | 0.53 | 1.3 | 0.6-2.6 |
| Other areas in Hubei province | 57 | 20.1 | 64 | 22.3 | 0.44 | 1.4 | 0.6-2.9 | 58 | 25.6 | 63 | 18.3 | 0.71 | 0.9 | 0.4-1.9 |
| Other provinces | 19 | 6.7 | 19 | 6.6 | — | — | — | 16 | 7.0 | 22 | 6.4 | — | — | — |
| Living in urban areas (vs rural) | 270 | 95.1 | 273 | 95.1 | 0.86 | 1.1 | 0.5-2.4 | 211 | 93.0 | 332 | 96.5 | 0.08 | 2.1 | 0.9-4.7 |
| Frequent use of mass media | 236 | 83.1 | 249 | 86.8 | 0.21 | 1.4 | 0.8-2.2 | 185 | 81.5 | 300 | 87.2 | 0.08 | 1.5 | 0.9-2.5 |
| Financial loss due to COVID-19 |  |  |  |  |  |  |  |  |  |  |  |  |  |  |
| None or minimal | 75 | 26.4 | 53 | 18.5 | — | — | — | 65 | 28.6 | 63 | 18.3 | — | — | — |
| Moderate | 169 | 59.5 | 168 | 58.5 | 0.32 | 1.2 | 0.8-1.9 | 133 | 58.6 | 204 | 59.3 | 0.13 | 1.4 | 0.9-2.1 |
| Significant | 40 | 14.1 | 66 | 23.0 | 0.004 | 2.3 | 1.3-4.0 | 29 | 12.8 | 77 | 22.4 | 0.004 | 2.4 | 1.3-4.2 |
| Poor or fair health perception (vs good) | 85 | 29.9 | 163 | 56.8 | <0.001 | 3.4 | 2.3-4.8 | 68 | 30.0 | 180 | 52.3 | <0.001 | 2.7 | 1.9-4.0 |
| Abbreviations: SD: standard deviation; OR: odds ratio; CI: confidential interval. | | | | | | | | | | | | | | |
